# Tetanus in a rural low-income intensive care unit setting

**DOI:** 10.1093/braincomms/fcab013

**Published:** 2021-02-16

**Authors:** Sam Olum, Jacob Eyul, Daniel Ocen Lukwiya, Neil Scolding

**Affiliations:** 1 Gulu University Faculty of Medicine, Gulu, Uganda; 2 St. Mary’s Hospital Lacor, Gulu, Uganda; 3 Institute of Clinical Neurosciences, University of Bristol, Bristol, UK

**Keywords:** tetanus, uganda, low-income setting

## Abstract

Tetanus is a potentially severe but preventable infection. In resource-rich settings, vaccination programmes have reduced tetanus to a rare disease, though still carrying an overall mortality of some 13%. However, in low-income settings, tetanus remains common, and is a significant cause of mortality—though major World Health Organisation programmes are successfully targeting neonatal and maternal disease. Data concerning the frequency and outcomes of non-neonatal tetanus in low-income settings are very sparse. We aimed to utilize a unique intensive care unit-based dataset to elicit clinical and demographic features and mortality in a large cohort of tetanus patients admitted over an eleven-year period to a single hospital centre in a rural low-income setting in northern Uganda. A total of 268 patients with tetanus were admitted to the Intensive Care Unit at St Mary’s Hospital, Lacor between 2005 and 2015; the records of 190 were retrievable and had sufficient information to be assessed. 29 were neonates (median age 7 days, IQR 0), 52 children (1–16yrs; median age 11 years, IQR 4.5) and 109 were adults (median age 42 years, IQR 23). There was no seasonal pattern in the frequency of admissions. Of the 190 patients, 69 had endotracheal intubation with intermitent positive pressure ventilation, and 57 patients had central line placement. The overall mortality was 51.5–72.4% in neonatal disease, 25% in children and 57.8% in adults. The requirements for neither central line insertion, nor endotracheal intubation, nor intermittent positive pressure ventilation were independently linked to mortality rates. By contrast with neonatal and childhood disease, there was a marked male preponderance in adult tetanus—94 males and 15 females (gender difference *P* < 0.001)—and although year-on-year breakdown suggested no obvious upward or downward trend over the span of our study in total numbers of tetanus admissions, a trend towards an increasing incidence of adult tetanus was apparent.These findings confirm that adult tetanus remains a major problem in rural low-income settings, particularly in males, and suggests that more resources should be devoted to vaccination programmes targeting men.

## Graphical Abstract

**Graphical Abstract:**
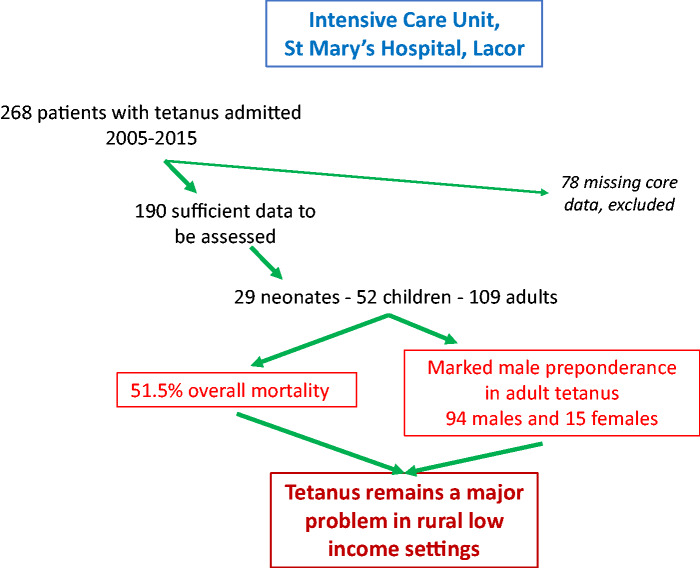

## Teaser

Tetanus is a severe but preventable infection. Olum *et al.* studied 190 tetanus admissions to one northern Uganda hospital. The overall mortality was 51.5%, with a marked male preponderance among adults—94 males versus 15 females. Their findings re-emphasise adult tetanus as a major problem in rural low-income settings.

## Introduction

Tetanus is an infection caused by the organism *Clostridiumtetani*, which is found in the soil and in the faeces of animals (including man) (Farrar *et al.*, 2000). Tetanus is usually the result of bacteria infecting a wound: deep penetrating wounds are particularly susceptible—crush injuries, for example—but tetanus can also follow superficial and otherwise trivial and unnoticed wounds (Farrar *et al.*, 2000; MEDSCAPE, 2017; Yen and Thwaites, 2019). *C.tetani* bacteria produce potent neurotoxins that cause involuntary and uncontrolled painful muscle contractions (Farrar *et al.*, 2000; CDC, 2017; Yen and Thwaites, 2019). These can be severe, and accompanied by respiratory compromise and autonomic instability; infection carries a high mortality rate.

Tetanus infection is preventable by vaccination; in resource-rich settings, vaccination programmes have reduced tetanus to a rare disease, though still carrying a mortality of some 13% (higher in those over 60 years or with diabetes mellitus) (CDC, 2011; Yen and Thwaites, 2019). However, in low- and middle-income countries, tetanus remains common, and is a major cause of mortality—estimated to be responsible for over 55000 deaths globally in 2015 (Kyu *et al.*, 2017), though relatively few published data are available.

St. Mary's Hospital Lacor is a 476 bedded not-for-profit private hospital serving approximately 700000 inhabitants of Gulu district and other areas of Northern Uganda, an area ravaged by conflict for over 20 years until approximately a decade ago. The region borders South Sudan, which still experiences major internal conflict. This has posed a particular problem to Gulu district as a result of compromised health provision including vaccination services.

A 2017 St. Mary’s Hospital survey of all Intensive Care Unit (ICU) admissions—5147 patients, medical, surgical, paediatric and obstetric, over some ten years—noted 37 infants with tetanus, in whom the mortality was almost 78% (Dunser *et al.*, 2017). The Lacor ICU database represents a unique clinical and research resource, not least in the current context since all tetanus cases at Lacor are admitted to ICU. We aimed to utilize this dataset more comprehensively, looking at adults and infants, to elicit clinical and demographic features, outcomes, and if possible key determinants of mortality in an unusually large cohort of tetanus patients admitted over an eleven-year period to a single hospital centre in a rural low-income setting.

## Methods

### Patient records

All patients presenting to St Mary’s Hospital Lacor, Gulu, Uganda, in whom a clinical diagnosis of tetanus is made, are admitted directly to the ICU. This Unit has maintained an extensive database recording all patients’ details since approximately 2004, thanks principally to the efforts of Dr Ray Towey (Towey and Ojara, 2008; Dunser *et al.*, 2017).

**Figure 1:**
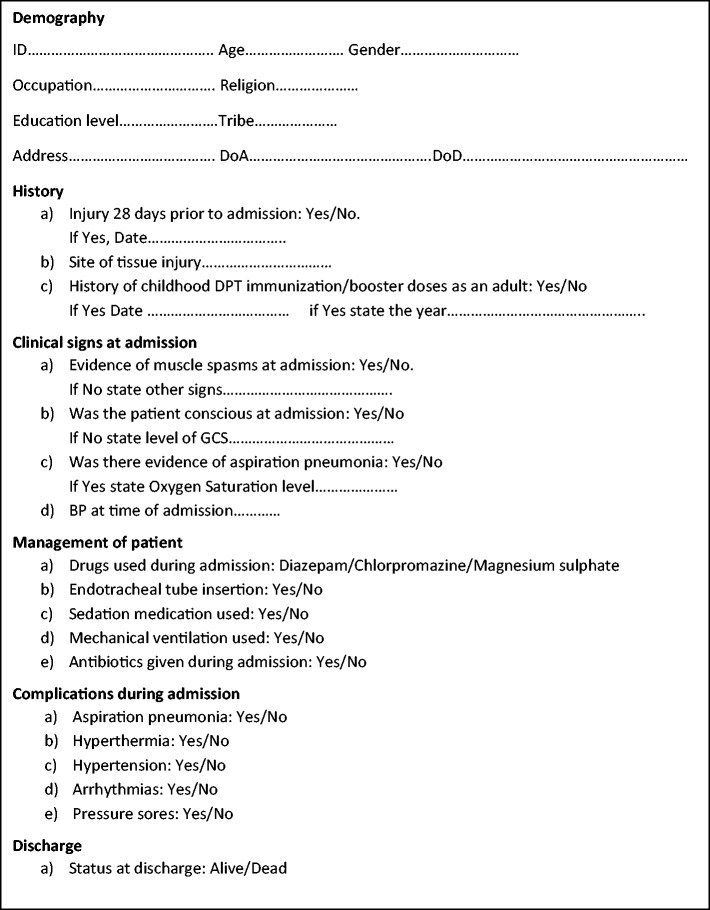
**Data Collection Tool.** Abbreviations: DoA = date of admission; DoD = date of discharge or death; DPT = diphtheria/pertussis/tetanus; GCS = Glasgow Coma Scale.

We utilized this database to conduct a retrospective cohort study looking at all tetanus patients treated in Lacor ICU from the year 2005 to 2015. A standardised and specifically-designed Data Collection Tool (Fig. 1) was used for collecting clinically relevant information.

Conventional paper records of all patients identified from the database were retrieved wherever possible and reviewed to check consistency and accuracy of electronic records and to glean additional clinical information. Some record sets were unavailable, others incomplete; we excluded from analysis all patients whose date of admission, gender or age, or discharge status could not be ascertained.

### Standard tetanus management at Lacor

All patients included in this study received care guided by either Lacor Hospital’s neonatal protocol or the adult protocol.

For neonates, this protocol included treatment with tetanus human immunoglobulin at a dose of 3000 IU intramuscularly. However, anti-tetanus serum was not readily available, and therefore no neonate received anti-tetanus serum. The protocol was, however, followed in the recommended use of diazepam 2 mg twice a day, clonazepam at 2.5–5.0 mg between 5–6 hourly and phenobarbitone at 10–15mg/kg body weight for breakthrough spasms.

The adult treatment plan included radical debridement and metronidazole 500 mg three times a day, tetanus human immunoglobulin at 3000–6000 IU, oxygen therapy as required, diazepam 10 mg–40mg, and magnesium sulphate (5 mg by intravenous infusion over 20 min). Nasogastric tube feeding was used, and tracheostomy for compromised airway and intermittent positive pressure ventilation with muscle relaxants as required. Again because of availability, only one adult in the study recieved the tetanus human immunoglobulin.

### Statistics

Data were entered and analyzed in STATA vs 10. Univariate and multivariate analysis were utilised as appropriate to determine the relationship and significance of various risk factors associated with tetanus or its outcome.

### Ethics

Ethical approval was obtained from the St Mary’s Hospital Lacor Institutional Review Board.

### Data availability

The data that support the findings of this study are available on request from the corresponding author. The data are not publicly available due to patient confidentiality issues.

## Results

### Total study population

A total of 268 patients with tetanus over the eleven-year period 2005–2015 were admitted to the ICU at St Mary’s Hospital, Lacor and had usable records. The records of 78 patients had missing core data and were therefore excluded from further analysis.

Of the remaining 190 patients, 23% were female and 77% male; 97 (51.5%) died. There was some gender difference in mortality—56.8% of females died compared to 49.3% of males, though this was not significant (*P* = 0.38).

Endotracheal intubation with intermittent positive pressure ventilation was administered to 69 patients, and 57 patients had central line placement. We explored whether the requirements on ICU for central line insertion, for endotracheal intubation, or intermittent positive pressure ventilation were independently linked to mortality rates; none were associated (data not shown).

**Figure 2:**
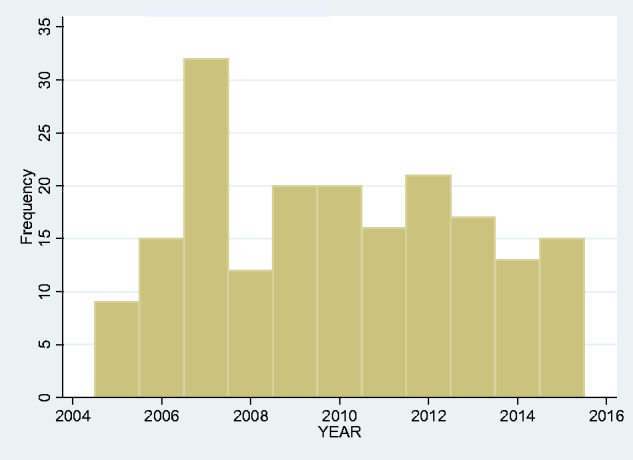
Yearly total incidence of tetanus 2005–2015.

**Figure 3:**
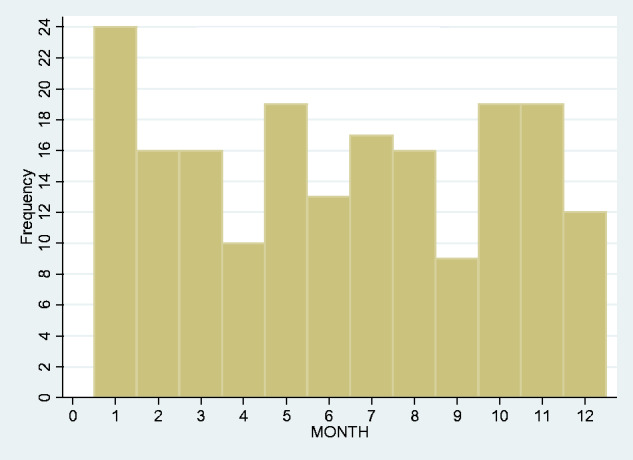
Incidence of tetanus by month.

We looked at the frequency of admissions year-by-year over the span of our study but found no obvious upward or downward trend (Fig. 2). We also looked at the monthly overall incidence (Fig. 3) and again found no overall seasonal pattern.

**Figure 4:**
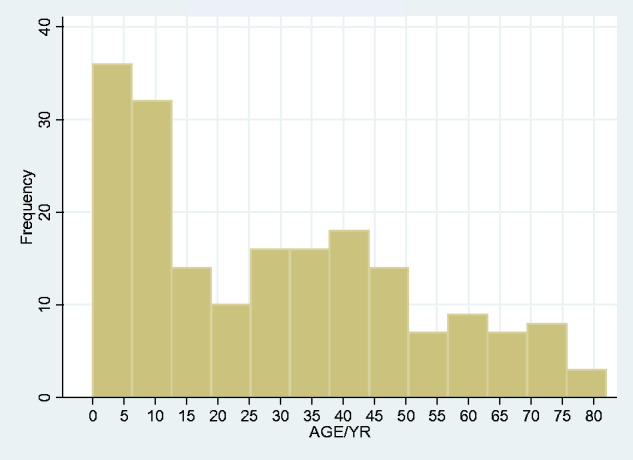
Age–incidence of all tetanus patients.

In age range, there was a clear peak in the neonatal and childhood range (Fig. 4). Given the obvious differences in particular in risk factors in neonatal disease compared to other age ranges, we looked at neonatal, childhood and adult onset tetanus populations separately.

### Neonatal tetanus (birth—10 days)

**Figure 5:**
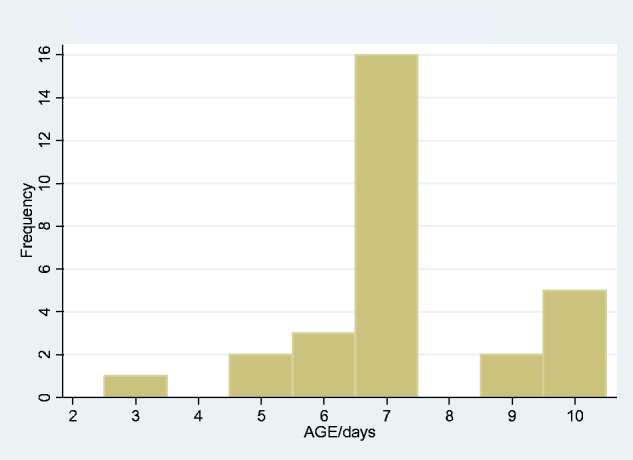
Age incidence of neonatal tetanus.

There were 29 neonatal cases of tetanus, 15 male and 14 female. The median age was 7 days, IQR 0. Of these 29, 21 died, an overall neonatal tetanus mortality of 72.4%. There was no significant difference in mortality according to gender (80% females died, 64.2% of males, *P* = 0.34). The peak age of onset was 7 days (Fig. 5). As with the total tetanus population, there was no changing trend of incidence over the data period, and no suggestion of seasonality in month of onset (data not shown).

### Childhood tetanus (1–16 years)

There were 52 individuals between 1 and 16 years of age, 14 females and 38 males. The median age was 11 years, IQR 4.5. (There were no recorded cases within the age bracket of 10 days to 1 year.) 13/52 died (25%), including 4 females (28.6%) and 9 males (23.7%). Yearly incidence did not vary significantly over the observation period; neither did average monthly incidence.

### Adult tetanus (17 years and over)

**Figure 6:**
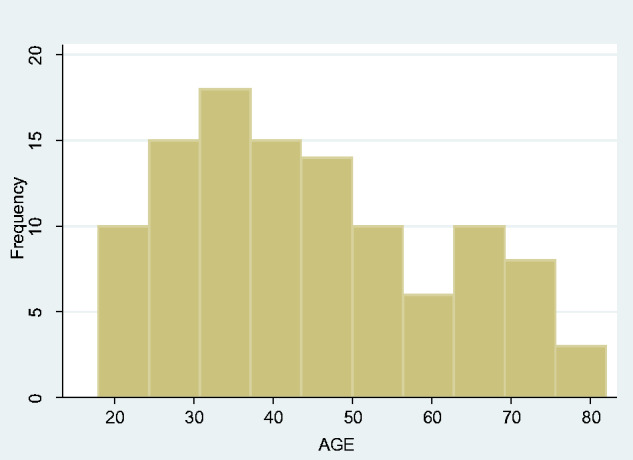
Age incidence of adult tetanus.

**Figure 7:**
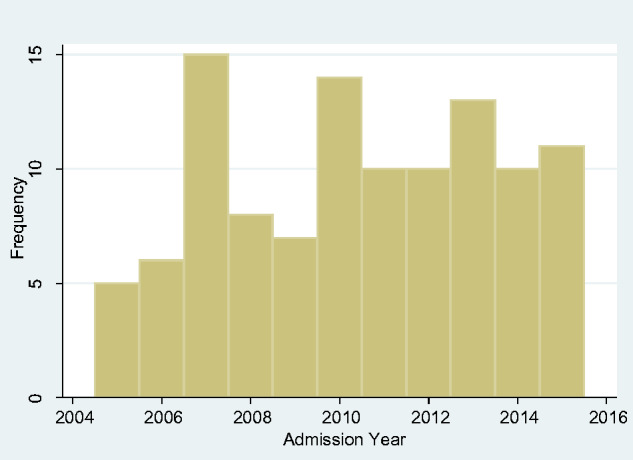
Yearly incidence of tetanus in adults 2005–2015.

There were 109 adults with tetanus admitted to St Mary’s Hospital Lacor ICU over the eleven-year period, with a striking male preponderance—94 males and 15 females (gender difference *P* < 0.001). The median age was 42 years, IQR 23. Of these, 63 (57.8%) patients died, 54 males (57.5%) and 9 females (60%). The age distribution appeared bimodal, with one peak at approximately 32 years and a second around 67 years of age (Fig. 6). There was no obvious seasonality (data not shown), but year-on-year breakdown suggested a trend towards an increasing incidence of adult tetanus (Fig. 7).

## Discussion

Our study provides unique insights into the pattern of tetanus occurrence in a rural LMIC setting and in particular into its associated mortality. Infant tetanus in such settings has been subject to much study in the past, but there are very few reports concerning adult disease. Although our study centres wholly on ICU patient records, we believe case ascertainment to be good: it has been known for some decades that the prognosis of tetanus is significantly improved by intensive care management (Trujillo *et al.*, 1987), and in consequence it is hospital policy at St Mary’s Lacor that all patients with tetanus are admitted to the ICU. Similarly, while there is a Government hospital in nearby Gulu, this has no ICU facility and so all tetanus patients will be transferred to Lacor.

A mean frequency of 17.3 cases each year in total in the known population of some 700000 allows the very crude estimation of a minimum incidence of 2.47/100000/year. In fact, however, if our total cohort were counted, i.e., including those whose demographic or clinical course data were incomplete, but whose diagnosis was secure, the figures become 24.4 cases per year, a truer minimum incidence of 3.5/100000/year. This compares with 0.007/100000/year in England (England, 2019). In Uganda in 2014, the incidence based on regional hospital returns was reported at approximately 9.3/100000 (Nanteza *et al.*, 2016). Incidence data for other LMICs are sparse and often imperfect (non-neonatal tetanus is not notifiable in many countries) but deaths are recorded: tetanus accounted for close to 25000 deaths in sub-Saharan Africa in 2015 (Kyu *et al.*, 2017)—or 2.5 deaths/100000.

**Figure 8:**
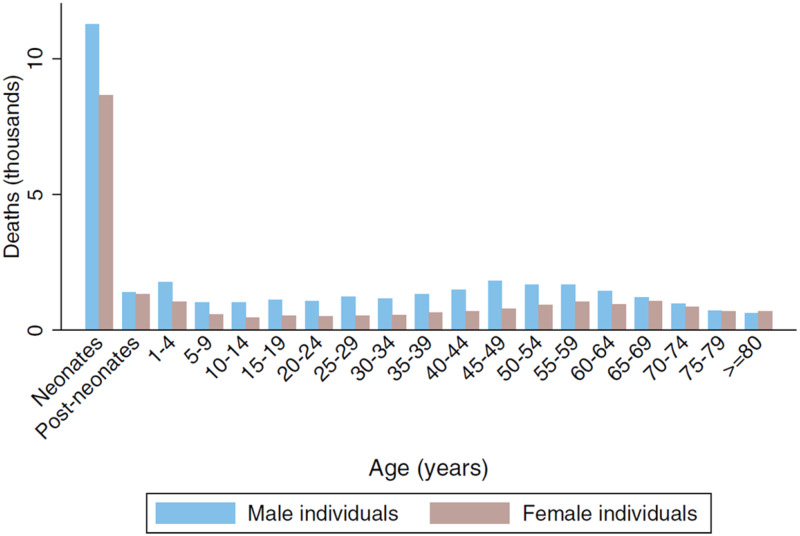
**Global age-sex distribution of tetanus deaths in 2015.** Reproduced with permission from Kyu et al. (2017).

85% of our patients were non-neonatal, yielding a population incidence of 2.1/100000—rather lower than the WHO crude estimate in 2013 of an incidence of 40/100000 non-neonatal cases across the whole African continent (Dalal *et al.*, 2016). The age-related incidence we found (Fig. 4), with a marked peak in the neonatal period and a less prominent second peak in adulthood, the median range 40–45 years, was not unexpected. Again, comparable data from other LMICs are not readily available, but our findings are in accord with the age-related incidence of deaths from tetanus (Fig. 8; reproduced with permission from Kyu *et al.* (2017)).

The overall mortality in our study was 51.1–58% in adults and 72% in neonates (and 25% in non-neonatal children). This is higher than the 30% recorded in the Ivory Coast (Tanon, 2017) (though 71% in the adult population), and the 33% in northern Ethiopia (Derbie *et al.*, 2016). In both latter studies, however, a significant number of patients discharged themselves from hospital against medical advice (over one third in the Ethiopian study)—with an expectation that the mortality amongst this group would be high (Derbie *et al.*, 2016). The only other, relatively small Ugandan study (25 cases) reported a case fatality of 47% (Nanteza *et al.*, 2016). In high-income countries, the hospital mortality was reported at approximately 13% in France (Yen and Thwaites, 2019), though in a Portuguese study the figure was between 32% and 59%, while in the USA in the over 60 s the mortality is over 50% (Farrar *et al.*, 2000).

Perhaps the most striking finding of our study was the substantial gender difference in incidence in adults. Within our total cohort of 190 patients, 23% were female and 77% male; but this included 29 neonatal cases of tetanus with an almost equal male: female division, 15 males and 14 females. In non-neonates, 82% were males, and of the 109 patients over the age of 16, 86% were male. Very few other LMIC data are available for disease incidence, but the global picture for tetanus deaths similarly stresses this imbalance. In two Ethiopian studies—one based in Addis (68 adults (Amare *et al.*, 2012)), one in north-west Ethiopia (106 adults (Derbie *et al.*, 2016)), 77.9% and 76.4% respectively were males. In an Ivory Coast study of 455 cases of non-neonatal tetanus, 85.7% were male (Tanon, 2017). A global WHO survey and literature study in 2016 found some 71% of patients admitted to hospital with tetanus were men (Dalal *et al.*, 2016), while a pan-African study of mortality found a female to male ratio a little less emphatic at 0.5 (Woldeamanuel *et al.*, 2016).

The reasons for this male preponderance are not entirely speculative: the WHO report (Dalal *et al.*, 2016) emphasised the global prioritization of eradicating maternal and neonatal tetanus launched by the World Health Assembly in 1988, with its successful emphasis on antenatal care, perinatal (and umbilical) hygiene and vaccination (Dalal *et al.*, 2016; Yen and Thwaites, 2019)—while the Addis Ababa study noted that, amongst the affected men, ‘none of them were vaccinated’(Amare *et al.*, 2012). An additional factor was the trend to circumcise adult men as a putative protection against AIDS transmission: this certainly caused tetanus in some individuals (Dalal *et al.*, 2016; Nanteza *et al.*, 2016). We did not have access to the vaccination records of our patients; in Uganda, children are vaccinated (DPT-Hep-Hib) at one month, six months, and one year; all females of reproductive age (15–45 years) receive booster doses (TT1; TT2—1 month; TT3—6 months; TT4—1 year; TT5—1 year), and those who do not will be offered vaccination during antenatal care (Uganda, 2017). This national programme was extended in 2005. There is no vaccination programme for males beyond childhood. Occasionally proposed as an explanation for the male preponderance but arguably less likely is the proposition that those undertaking agricultural work are most likely to be exposed to tetanus spores—since in many LMICs, women undertake much of the agricultural labour.

In summary, our study highlights the continuing high burden and fatality rate of tetanus in a rural sub-Saharan African setting—a disease difficult to treat even within resource-rich settings, but which is almost entirely preventable if not eradicable. Our findings re-emphasise the need to concentrate on adult males and to find ways of increasing uptake of vaccination. Finally, our data provide no suggestion of any decreasing incidence over the 11 year period to 2015—contrasting with the global picture of an 80% fall in tetanus-associated mortality between 1990 and 2005 (Kyu *et al.*, 2017). Rather, year-on-year breakdown of adult tetanus in northern Uganda suggested a trend towards an increasing incidence.

## Funding

NJS was supported by a grant from The Burden Trust.

## Competing Interests

The authors report no competing interests.

## Abbreviations

ICU =: intensive care unit
LMIC =: low or middle-income country
WHO =: world health organisation
